# Anti-tumor effect of antibody drug conjugate ASP1235 targeting Fms-like tyrosine kinase 3 with venetoclax plus azacitidine in an acute myeloid leukemia xenograft mouse model

**DOI:** 10.18632/oncotarget.28331

**Published:** 2022-12-20

**Authors:** Hirofumi Tsuzuki, Tatsuya Kawase, Taisuke Nakazawa, Masamichi Mori, Taku Yoshida

**Affiliations:** ^1^Immuno-Oncology, Astellas Pharma Inc., Tsukuba, Ibaraki 305-8585, Japan; ^2^Applied Research and Operations, Astellas Pharma Inc., Tsukuba, Ibaraki 305-8585, Japan

**Keywords:** acute myeloid leukemia (AML), ASP1235, antibody drug conjugate (ADC), venetoclax, azacitidine

## Abstract

Antibody drug conjugates (ADC) are one of the attractive modalities for the treatment of acute myeloid leukemia (AML). Previously, we have developed ASP1235, a novel ADC targeting Fms-like tyrosine kinase 3 (FLT3) which is widely expressed on the leukemic blasts of AML patients. In this study, we sought to evaluate the therapeutic effect of ASP1235 in combination with venetoclax plus azacitidine, a novel standard-of-care treatment for elderly AML patients, in ASP1235 poor sensitive AML cells. To identify the suitable preclinical model, we first evaluated the growth inhibitory effect of ASP1235 on several leukemia cell lines expressing FLT3 and found that THP-1 cells were partially sensitive to ASP1235 *in vitro*. Furthermore, ASP1235 showed marginal anti-tumor activity in a THP-1 xenograft model. Compared to the leukemic blasts in most of the relapsed or refractory (R/R) AML patients tested, THP-1 cells expressed equivalent protein levels of Bcl-2, suggesting that ASP1235 in combination with venetoclax plus azacitidine is a rational treatment in the THP-1 model. *In vitro*, ASP1235 showed a cytotoxic effect on THP-1 cells in combination with venetoclax, and the combination effect was greater than the additive effect. Furthermore, ASP1235 also showed a combination effect with venetoclax plus azacitidine treatment. Similarly, the combination of ASP1235, venetoclax and azacitidine showed a superior anti-tumor effect in a THP-1 xenograft model without obvious body weight loss. These findings provide supportive evidence that the triple combination of ASP1235, venetoclax and azacitidine would improve the clinical outcome of ASP1235 monotherapy and venetoclax plus azacitidine regimen in AML patients.

## INTRODUCTION

Acute myeloid leukemia (AML) is an aggressive hematologic malignancy associated with various cytogenetic and molecular abnormalities in myeloid lineage leukemic cells [1]. Predictions suggest that in 2021, approximately 20,240 people will be newly diagnosed with AML, and 11,400 AML patients will die from the disease in the United States [2]. Over the last four decades, the frontline therapy of AML patients under 60 years old consists of intensive chemotherapy such as cytarabine and anthracycline-based regimen, followed by allogenic hematopoietic stem cell transplantation (allo-HSCT) and/or consolidation chemotherapy. In spite of the promising clinical response, patients eventually relapse, resulting in only 40% survival during 5 years [3]. Older AML patients do not tolerate the treatment mentioned above, hence they are subjected to chemotherapy with lower intensity such as low-dose cytarabine, azacitidine or decitabine. However, the median overall survival of these patients is under one year [3]. Recently, the combination treatment of azacitidine plus venetoclax, a selective inhibitor for an anti-apoptotic protein Bcl-2 that is overexpressed and is a critical cause of drug resistance in AML [4, 5], showed promising clinical response and prolonged the survival of AML patients compared to the azacitidine monotherapy treated group [6], resulting in approval by the Food and Drug Administration. However, some patients did not reach complete remission [6] and many patients ultimately relapse after the combination treatment of venetoclax plus azacitidine [7]. Therefore, unmet medical needs still exist in the treatment of AML. Given that the median age of AML patients is 67 years old [8], the development of a novel drug with high efficacy and lowered toxicity is strongly preferred.

Antibody drug conjugates (ADC) are one of the modalities that aims to dissociate drug efficacy from toxicity. ADC consists of three components: antibody specific for tumor associated antigen, drug linker and cytotoxic payload. By the specific binding to tumor cells through the antibody, the cytotoxic payload is accumulated in the tumor tissue, resulting in anti-tumor efficacy with minimized toxicity against normal tissues [9]. Some ADCs have already been approved and used for the treatment of many types of cancers with promising clinical outcomes [9]. In terms of AML, gemtuzumab ozogamicin (GO), an ADC targeting CD33, has been re-approved and applied as a frontline therapy for CD33-positive AML patients, demonstrating the proof of clinical benefit of ADC in AML treatment [10]. However, there are some limitations with the use of GO due to drug resistance such as efflux of the payload calicheamicin via P glycoprotein (P-gp), an ATP-driven transporter for hydrophobic drugs, and the unresponsiveness of patients with low CD33 positivity [11]. Therefore, the development of a novel ADC is required to overcome these problems.

Previously, we have developed a novel ADC, ASP1235 which was originally named AGS62P1, targeting Fms-like tyrosine kinase 3 (FLT3) which is overexpressed on the leukemic blasts in over 90% of AML patients [12, 13]. AGL-0182-30, a newly developed microtubule disrupting agent, is linked to the antibody of ASP1235 as a payload, and we previously showed that this payload is poorly effluxed from cancer cells via P-gp [12]. We also showed that AGL-0182-30 or the metabolite showed tumor growth inhibition of AML cell lines *in vitro*, indicating that ASP1235 shows anti-tumor effect through releasing its cytotoxic payload in AML cells [12]. The clinical outcome of ASP1235 monotherapy on R/R AML patients has been evaluated at a phase 1 clinical study (NCT02864290).

Recent studies suggested that the efficacy of ADC would be improved by combination treatment with other classes of drugs including standard chemotherapy [14], which would provide the patients with better clinical outcome. In the context of AML, as mentioned above, the venetoclax plus azacitidine regimen is a promising standard care treatment used in elderly patients with AML. Thus, in this study, we sought to evaluate the combination effect of ASP1235 with venetoclax plus azacitidine regimen by utilizing a suitable and clinically relevant preclinical model.

## RESULTS

### ASP1235 partially inhibited the growth of THP-1 cells both *in vitro* **and** *in vivo*

**Figure 1:**
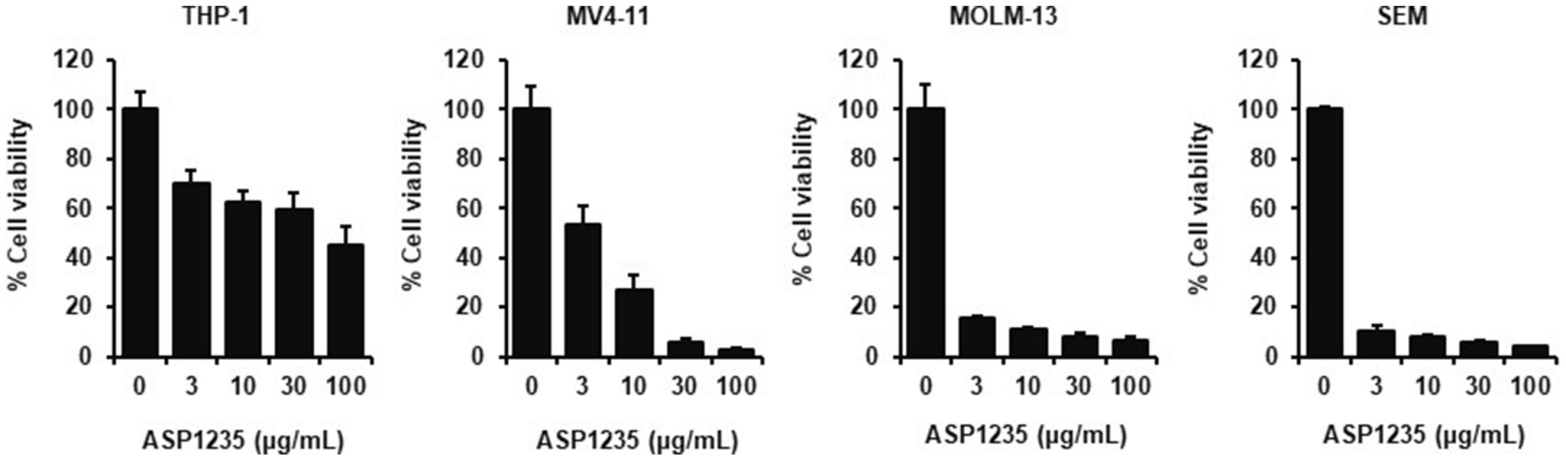
Growth inhibitory effect of ASP1235 in leukemia cells. AML cell lines or SEM cells were treated with ASP1235 at the indicated concentrations for 3 days. Cell viability was measured as described in Materials and Methods. Data are shown as means ± standard deviation of the triplicate.

To identify a preclinical model suitable for evaluating the combination treatment of ASP1235 with venetoclax plus azacitidine, we investigated the growth inhibitory effect of ASP1235 on several leukemia cell lines *in vitro*. ASP1235 well suppressed the growth of AML cell lines such as MV4-11 and MOLM-13, and SEM, an acute lymphoblastic leukemia cell line (Figure 1). On the other hand, the sensitivity of ASP1235 on an AML cell line THP-1 was partial with around 40% of cells alive even at 100 μg/mL (Figure 1).

**Figure 2:**
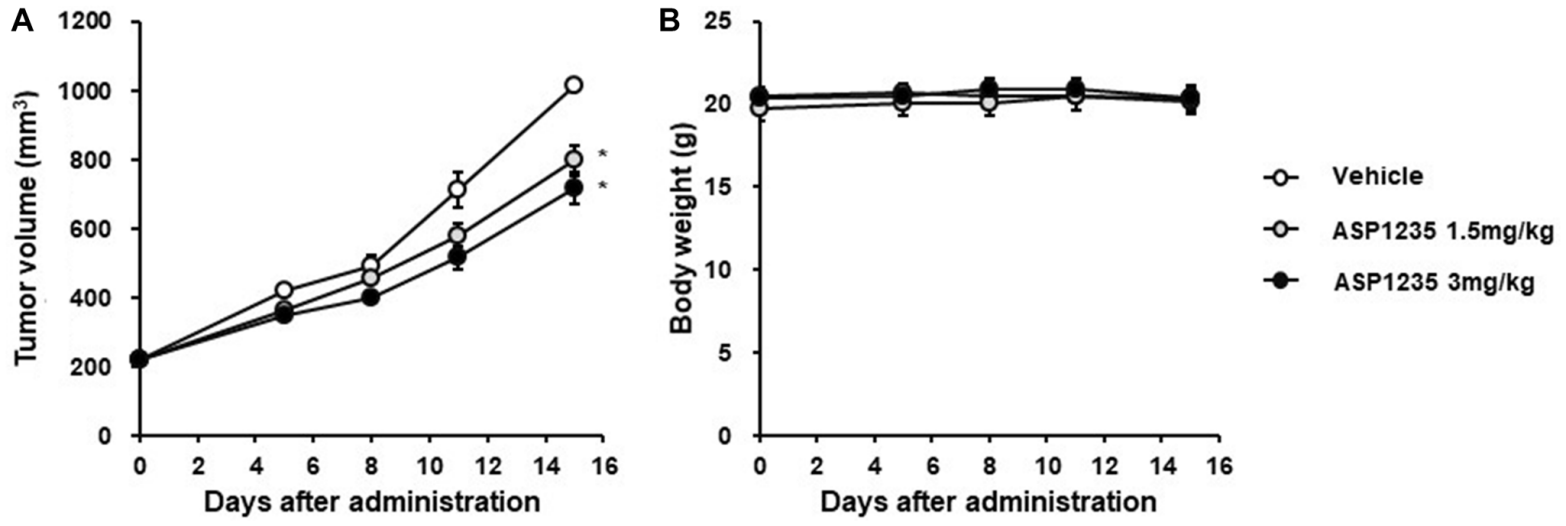
ASP1235 partially inhibited the tumor growth in a THP-1 xenograft mouse model. THP-1 xenograft mice were intraperitoneally injected 20 mg/kg of nonspecific human IgG1 antibody on day 0 and day 7. ASP1235 with the indicated dose was intravenously injected on day 1 and day 8. The means ± standard errors of (**A**) tumor volume and (**B)** body weight are shown (*n* = 6/group). Dunnett’s multiple comparison test was used for the statistical analysis. ^*^ means *P* < 0.05.

Next, we investigated the *in vivo* anti-tumor effect of ASP1235 using THP-1 xenograft mouse model. ASP1235 at 1.5 and 3 mg/kg significantly inhibited the tumor growth by 27% and 38%, respectively (Figure 2). These results indicate that THP-1 cell is a partial sensitive preclinical model to ASP1235.

### FLT3 and Bcl-2 expression in THP-1 cells are similar to those in primary leukemic blasts from chemotherapy R/R AML patients

**Figure 3:**
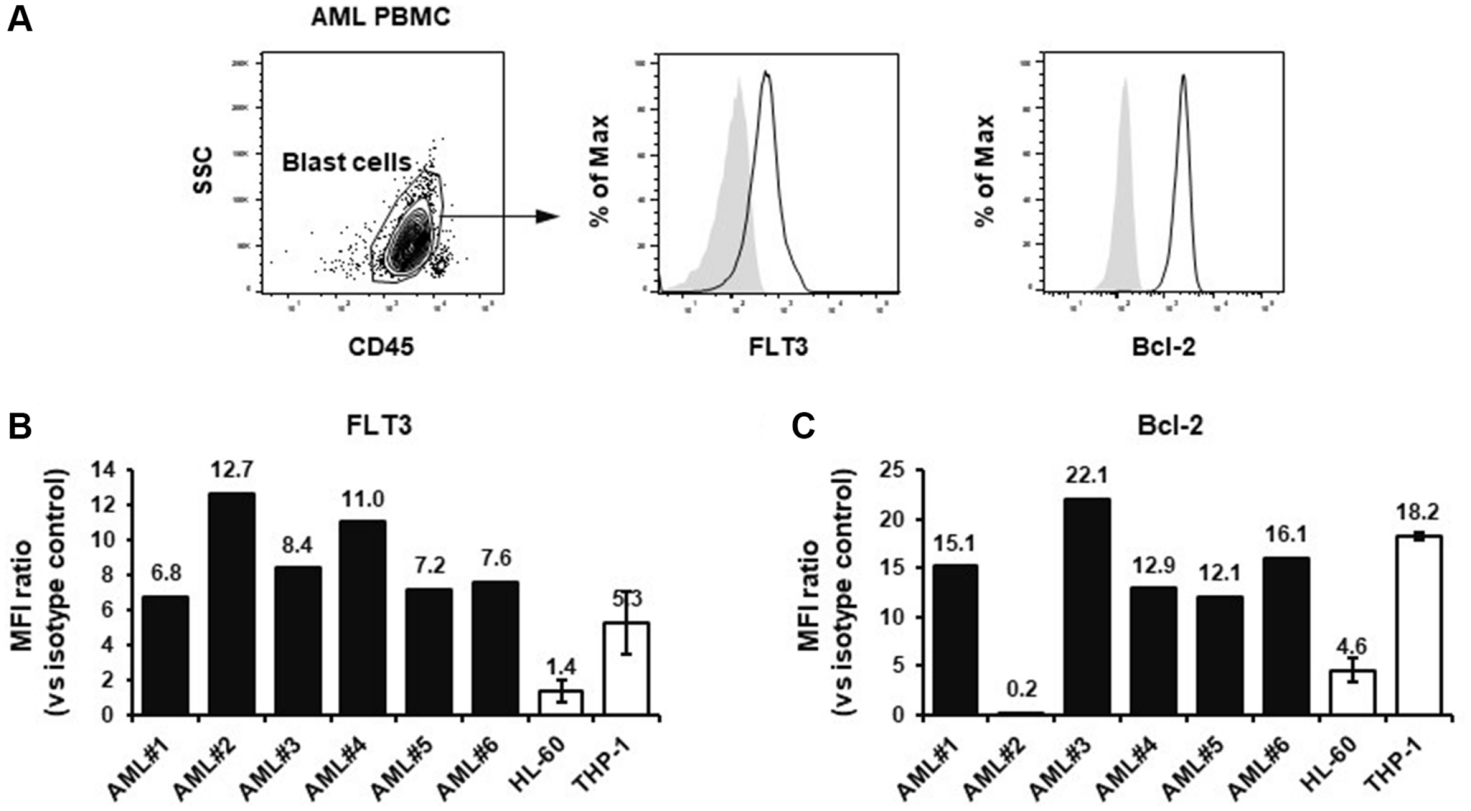
FLT3 and Bcl-2 expression in THP-1 cells and PBMCs from chemotherapy relapsed or refractory AML patients. (**A**) The gating strategy of AML blast population is shown. The geometric mean fluorescence index (MFI) ratios (vs. isotype control) of (**B**) FLT3 and (**C**) Bcl-2 are shown. In terms of HL-60 and THP-1 cells, the means ± standard deviations of three independent measurements are shown.

It has been reported that the proportion of patients showing high Bcl-2 expression was greater in chemotherapy R/R AML patients compared to that in newly diagnosed patients [4]. Thus, we investigated the expression levels of Bcl-2 together with FLT3 to further consider the relevance of THP-1 cells for evaluation on the combination treatment with venetoclax. We compared the expression levels of FLT3 and Bcl-2 in THP-1 cells with those of primary leukemic cells isolated from chemotherapy R/R AML patients to consider the clinical relevance on the expression levels. The gating strategy of leukemic blast population in the PBMCs of AML patients is shown in Figure 3A. Briefly, the leukemic blast fraction was defined as SSC^low^ CD45^dim^ population in PBMC. THP-1 cells expressed FLT3 at a level similar to that on primary AML cells, which are thought to express high levels of FLT3 (Figure 3B). As for Bcl-2, the expression level in THP-1 cells was also relatively comparable to that in most of the leukemic blasts of R/R AML patients tested (Figure 3C). Therefore, the expression levels of FLT3 and Bcl-2 of THP-1 cells were found to be clinically relevant, and we speculated that the anti-tumor effect of ASP1235 could be potentiated by the combination treatment with venetoclax plus azacitidine regimen, a novel standard-of-care treatment for elderly patients with AML.

### ASP1235 showed enhanced cytotoxic effect on THP-1 cells in combination with venetoclax or venetoclax plus azacitidine *in vitro*

**Figure 4:**
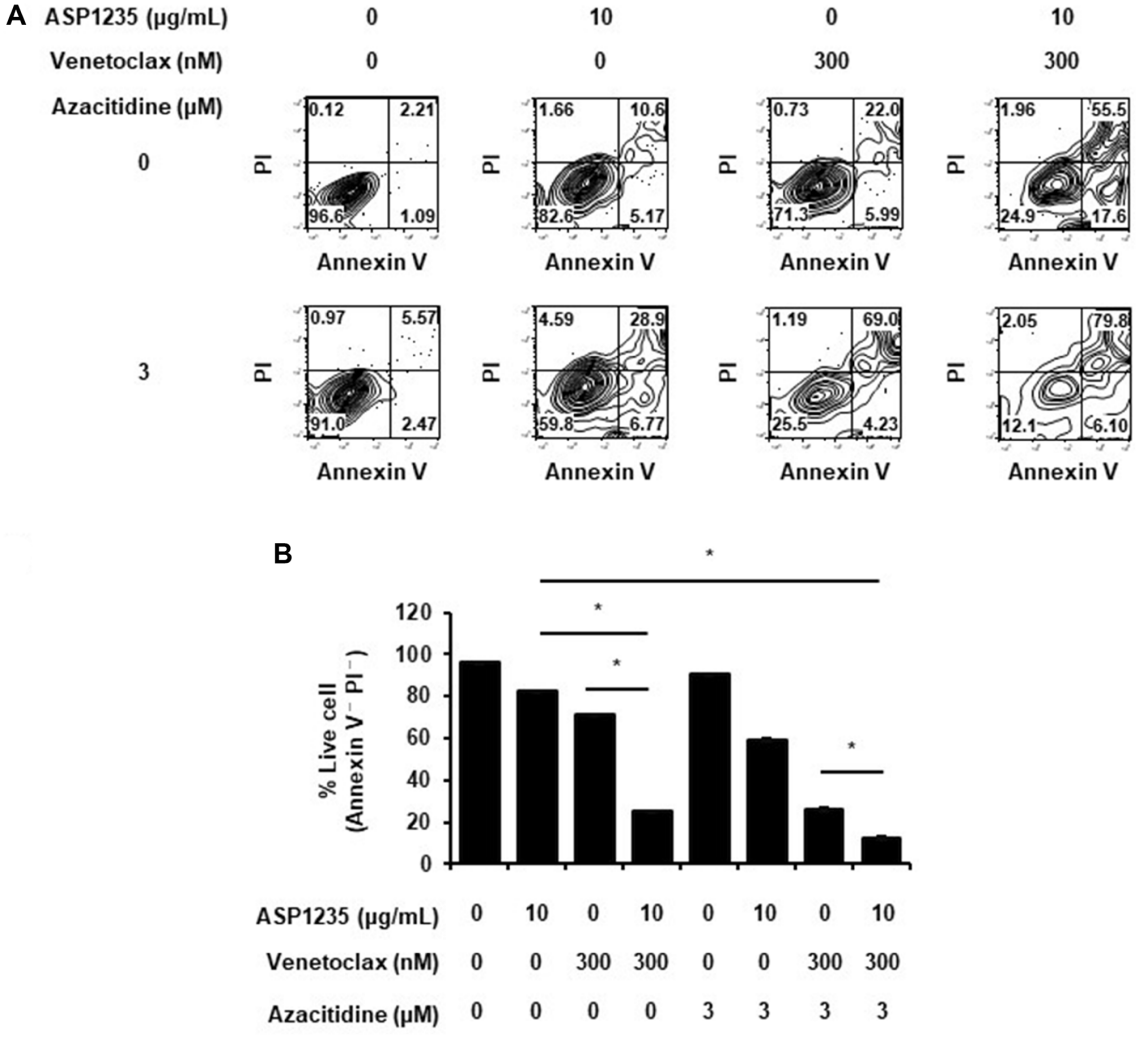
ASP1235 showed enhanced cytotoxic effect with venetoclax or venetoclax plus azacitidine *in vitro*. THP-1 cells were treated with or without ASP1235, venetoclax and azacitidine at the indicated concentrations. (**A**) Representative plots of annexin V - propidium iodide (PI) assay are shown. (**B**) Representative data from at least two independent experiments are shown. Data are shown as mean ± standard deviation of the triplicate. ^*^ means *P* < 0.05.

To confirm our hypothesis that the effect of ASP1235 would be sensitized by the combination treatment with venetoclax plus azacitidine, we first performed *in vitro* apoptosis assay using THP-1 cells. As expected, ASP1235 showed combination cytotoxic effect with venetoclax on THP-1 cells, and the combination effect was greater than additive effect (Figure 4). Given that the combination treatment of venetoclax and azacitidine has already showed promising anti-leukemic effect in AML patients [6], we next examined whether ASP1235 shows combination effect with the treatment of venetoclax plus azacitidine. The triple combination of ASP1235, venetoclax and azacitidine showed significant superior efficacy to ASP1235 single agent or venetoclax plus azacitidine treatment. Similar results were also obtained with MV4-11 cells sensitive to ASP1235 (Figure 1, Supplementary Figure 1).

### ASP1235 showed enhanced *in vivo* anti-tumor effect in combination with venetoclax plus azacitidine in THP-1 xenograft mouse model

**Figure 5:**
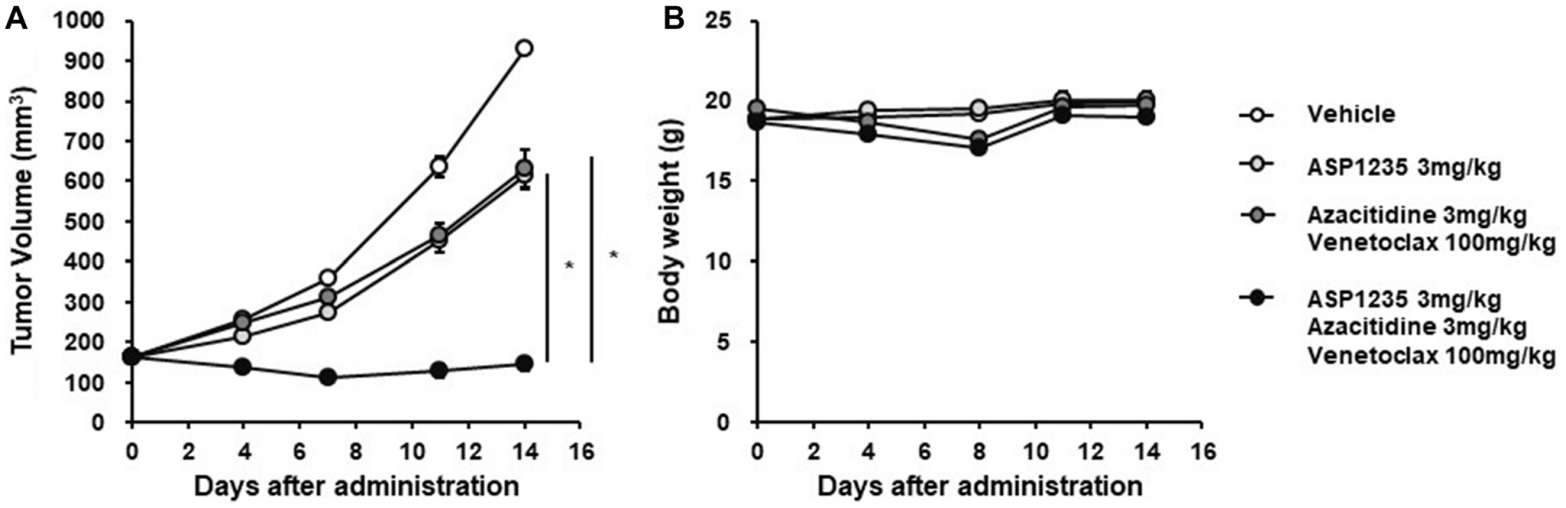
ASP1235 showed enhanced anti-tumor effect in combination with venetoclax and azacitidine in THP-1 xenograft mouse model. THP-1 xenograft mice were intraperitoneally injected 20 mg/kg of nonspecific human IgG1 antibody on day 0 and day 7. ASP1235 with the indicated dose was intravenously injected on day 1 and day 8. Venetoclax was orally administered daily. Azacitidine was intravenously injected for 5 consecutive days from day 1 to day 5. The means ± standard errors of (**A**) tumor volume and (**B**) body weight are shown (Vehicle group, ASP1235 group, azacitidine plus venetoclax group: *n* = 8/group, triple combination group: *n* = 7/group). Student’s *t*-test was used for the statistical analysis. ^*^ means *P* < 0.05.

We next investigated whether the triple combination treatment of ASP1235, venetoclax and azacitidine shows benefit *in vivo* using the THP-1 xenograft mouse model. Consistent with *in vitro* observations in Figure 4, triple combination treatment with ASP1235, venetoclax and azacitidine induced tumor regression, and the anti-tumor effect of the triple combination was much stronger than that of ASP1235 single agent or venetoclax plus azacitidine without obvious body weight loss (Figure 5). Given that relapse or lack of response after venetoclax plus azacitidine treatment is of emerging clinical significance, the combination therapy of ASP1235, venetoclax and azacitidine would be beneficial for AML patients to improve clinical outcomes.

## DISCUSSION

In this report, we evaluated the anti-tumor effect of ASP1235 in combination with venetoclax or venetoclax plus azacitidine for THP-1 cells, which are AML-derived cells expressing both FLT3 and Bcl-2. As expected, the combination treatment induced apoptosis more efficiently compared to each treatment alone *in vitro*. The triple combination showed superior anti-tumor efficacy with tumor regression to ASP1235 alone or venetoclax plus azacitidine. These findings suggest that the clinical efficacy of ASP1235 would be improved by the combination with venetoclax and azacitidine. In the past decades, evidence demonstrated that Bcl-2 signaling is a critical cause of chemotherapy resistance. In the setting of AML, Bcl-2 expression is elevated in the leukemic cells of AML patients compared to that of normal PBMCs [4]. Interestingly, it has been reported that the proportion of patients showing high Bcl-2 expression was greater in chemotherapy R/R AML patients compared to that in newly diagnosed patients [4]. In terms of prognosis, AML patients with high Bcl-2 positivity showed poorer prognosis than those with Bcl-2 low positivity following intensive chemotherapy treatment [15]. As mentioned above, in a recent phase III clinical trial with venetoclax and azacitidine combination treatment in AML patients, it has been demonstrated that venetoclax increased the sensitivity of azacitidine and improved the clinical outcome of AML patients [6]. These findings suggest that the effect of chemotherapy could be potentiated by the combination with venetoclax in AML. Given that ADC shows anti-tumor effect through the cytotoxic payload conjugated to the antibody, we thought that the same logic could be true of ASP1235. Again, in this study, we demonstrated that the sensitivity of ASP1235 was increased by the combination treatment with venetoclax or venetoclax plus azacitidine treatment both *in vitro* and *in vivo*. Therefore, we expect that the triple combination treatment of ASP1235, venetoclax and azacitidine would improve the clinical outcome of ASP1235 in AML patients.

Recently, the combination treatment of venetoclax with azacitidine showed promising clinical response in AML patients, however, the resistance and relapse from the combination treatment became clear. As for the clinical benefit of venetoclax plus azacitidine, evidence has been reported recently. In the phase II clinical trial, the overall response rate (ORR) of venetoclax monotherapy in AML patients was 19% [16]. And, the ORR of azacitidine or decitabine monotherapy was 25% in R/R AML patients [17]. On the other hand, in a retrospective analysis, the ORR of the combination treatment of venetoclax with azacitidine or decitabine was 64% [18], indicating a significant improvement in the clinical response compared to that in each monotherapy. Actually, in a recent phase III clinical trial, venetoclax plus azacitidine treatment significantly prolonged the survival of newly diagnosed AML patients ineligible for high intensity induction chemotherapy compared to the azacitidine monotherapy treated group [6], which is a promising clinical outcome. However, some patients did not reach complete remission in this trial, and it has been reported that about half of the AML patients ultimately relapse after the venetoclax plus azacitidine combination treatment [7]. Therefore, unmet medical needs still exist to improve the clinical outcome of venetoclax plus azacitidine. In this study, we showed that THP-1 xenograft mouse model was poorly sensitive to venetoclax plus azacitidine treatment (Figure 5A), which might mimic the AML patients less sensitive to the combination treatment. On the other hand, the triple combination treatment with ASP1235, venetoclax and azacitidine induced tumor regression in the THP-1 xenograft model, and the anti-tumor effect was greater than that of venetoclax plus azacitidine (Figure 5A). These findings strongly suggest that the triple combination of ASP1235, venetoclax and azacitidine could improve the clinical outcome of not only ASP1235 monotherapy but also venetoclax plus azacitidine treatment in AML patients. And, as for the safety profile, there was no obvious body weight loss caused by the triple combination treatment in this study, thus we expect the treatment would be tolerable for patients, which should be carefully investigated. Future clinical investigation is warranted.

Considering the whole therapeutic algorithm of AML, the progress of drug development for AML patients unfit for molecular targeted therapies (MTTs) is relatively poor, and we believe that the triple combination treatment of ASP1235, venetoclax and azacitidine provides a benefit for AML patients including these cases. Recent advances in the development of MTTs targeting driver mutations prolonged the survival of AML patients. For example, FLT3 mutations such as point mutations in the tyrosine kinase domain (TKD) and internal tandem duplication (ITD) in the jaxtamembrane (JM) domain are observed in about 30% of AML patients and the patients harboring these FLT3 mutations show poor prognosis [19]. Tyrosine kinase inhibitors for mutant type FLT3 such as gilteritinib, quizartinib and midostaurin efficiently suppress the growth of FLT3 mutation positive AML cells in preclinical studies and prolong survival of AML patients in clinical trials [20]. For instance, in a phase 3 clinical trial on FLT3 mutation positive R/R AML patients, gilteritinib single agent significantly prolonged survival: the median overall survival (OS) was 9.3 months in the gilteritinib treatment group, while 5.6 months in the standard chemotherapy treatment group [21]. Recently, to maximize the clinical benefit of these tyrosine kinase inhibitors, many clinical studies with combination treatments of FLT3 inhibitors and other drugs including standard chemotherapies are in progress, and combination benefit has already been reported for midostaurin [22]. Other than FLT3 mutation, many additional driver mutations are reported in AML patients with lower frequency [23], and the development of MTTs specific to these mutations are in progress [24]. On the other hand, unmet medical needs remain for AML patients unfit for MTTs. In this study, we showed that treatment with a triple combination of ASP1235, venetoclax and azacitidine significantly suppressed the growth of THP-1 cells, which harbor an Nras driver mutation that makes treatment with current MTTs unsuitable, both *in vitro* and *in vivo* (Figures 4 and 5). Therefore, we expect that the triple combination of ASP1235, venetoclax and azacitidine would be beneficial for FLT3 positive AML patients including cases unfit for MTTs in the clinic.

We speculate that there are many factors involved in the less sensitivity of ASP1235. In general, the expression level of tumor associated antigen is considered to be a predictive marker of the sensitivity of tumor cells to ADC. However, it has been reported that the expression level of FLT3 on THP-1 is higher than that on MV4-11 and MOLM-3 [25], while the sensitivity of ASP1235 on THP-1 was poorer compared to MV4-11 and MOLM-13 (Figure 1), suggesting that FLT3 expression level does not always reflect the cytotoxic activity of ASP1235 on leukemia cells. As for FLT3 mutation status, ASP1235 showed similar cytotoxic activity not only against MV4-11 and MOLM-13 cells which harbor FLT3 mutation but also against SEM cells which express wild type FLT3 (Figure 1). Therefore, we speculate that the cytotoxic activity of ASP1235 is not dependent on FLT3 mutation status. In terms of Bcl-2 expression, it has been reported that MV4-11 and MOLM-13 also express Bcl-2, while these cell lines were sensitive to ASP1235 single treatment (Figure 1). We think the total expression profile of the other apoptosis-related proteins would be involved in the sensitivity. For example, MV4-11 expressed higher level of pro-apoptotic protein Bax compared to THP-1 [26]. In addition, THP-1 expressed anti-apoptotic protein Mcl-1 higher than MV4-11 and MOLM-13 [26]. Given that these apoptosis-related proteins including Mcl-1 are involved in drug sensitivity of cancer cells [27], the balance of the apoptotic signal might be one of the important factors to determine the response to ASP1235 monotherapy. It would be worth testing if these apoptosis related proteins can be used as predictive markers of the drug response by using clinical samples.

Outside of AML, the combination treatment with ASP1235 and venetoclax might be beneficial in other types of cancers overexpressing FLT3. One example is B-acute lymphoblastic leukemia (B-ALL), which is a hematological malignancy arising from immature B cell precursor cells. Although over 80% of pediatric ALL patients including B-ALL reach long-term remission after the standard therapy, the 5-year overall survival of adult ALL patients remains about 20 to 40% [28], suggesting there are still unmet medical needs in this population. It has been reported that the leukemic blast cells in B-ALL patients highly express FLT3 [29, 30]. As for the contribution of Bcl-2 in the B-ALL, it has been reported that venetoclax single agent suppressed the growth of a B-ALL cell line or B-ALL patient-derived cells [31], suggesting that Bcl-2 contributes to the cell survival of B-ALL leukemic cells. In addition, venetoclax potentiated the effect of tyrosine kinase inhibitor, chemotherapy and the inhibitor for Mcl-1, an anti-apoptotic protein belonging to Bcl-2 family, when used in combination in a preclinical study [31]. Furthermore, about 60% of R/R ALL patients including B-ALL who received combination treatment of venetoclax with low-dose navitoclax, a Bcl-2/Bcl-XL inhibitor, reached complete remission in a phase 1 clinical trial (NCT03181126) [32]. These findings suggest that Bcl-2 also contributes to drug resistance in B-ALL and Bcl-2 inhibition sensitizes the effect of cytotoxic drugs. Taken together, we expect that ASP1235 single agent and the combination treatment of ASP1235 and venetoclax regimen would also be beneficial for B-ALL patients. To this end, further preclinical and clinical studies are required.

In conclusion, the triple combination treatment of ASP1235, venetoclax and azacitidine has the potential to benefit AML patients, and there is a possibility to expect the combination effect of ASP1235 and venetoclax regimen in FLT3 positive cancers beyond AML.

## MATERIALS AND METHODS

### Reagents

ASP1235 was prepared at Astellas Pharma Inc. L-Histidine (Catalog # H8000-5G) and Polysorbate 20 (Tween 20, Catalog # 441122-100G-F) were purchased from Sigma-Aldrich (St. Louis, MO, USA). Venetoclax was purchased from LC Laboratories (Catalog # V-3579, Woburn, MA, USA) or Chemietek (Catalog # CT-A199, Indianapolis, IN, USA). Azacitidine was purchased from Tokyo Chemical Industry Co., Ltd (Catalog # A2033, Tokyo, Japan). L-Histidine-HCl was purchased from Kanto Chemical Co, Inc (Catalog # 18064-30, Tokyo, Japan). Trehalose dihydrate (Catalog # 204-18451), polyethylene glycol 400 (PEG400, Catalog # 161-09065) and ethanol were obtained from FUJIFILM Wako Pure Chemical Industries Ltd (Osaka, Japan). Phosal 50PG was purchased from H. Holstein Co., Ltd (Catalog # 368315, Tokyo, Japan).

### Animals

Four weeks old female CB17/Scid mice were purchased from Charles River Japan, Inc (Yokohama, Japan, RRID: not applicable) and used in the following *in vivo* study. All animal experiments were approved by the Institutional Animal Care and Use Committee in Astellas Pharma Inc. In addition, Tsukuba Research Center of Astellas Pharma Inc. was accredited by AAALAC International.

### Cell line and cell culture

THP-1 (Catalog # TIB-202, RRID: CVCL_0006) and MV4-11 (Catalog # CRL-9591, RRID: CVCL_0064) cells were purchased from American Type Culture Collection (Manassas, VA, USA), Inc. MOLM-13 (Catalog # ACC 554, RRID: CVCL_2119) and SEM (Catalog # ACC 546, RRID: CVCL_0095) cells were purchased from DSMZ-German Collection of Microorganisms and Cell Cultures GmbH (Braunschweig, Germany). HL-60 cells were purchased from European Collection of Authenticated Cell Cultures (Catalog # 98070106, Salisbury, United Kingdom, RRID: CVCL_0002). THP-1 and MOLM-13 cells were cultured in RPMI-1640 (Sigma-Aldrich, Catalog # R8758) supplemented with 10% fetal bovine serum (FBS) (GE Healthcare, Catalog # SH30084.03, Chicago, IL, USA). MV4-11 and SEM cells were cultured in Iscove’s modified Dulbecco’s medium (IMDM) (Catalog # 12440-053, ThermoFisher Scientific, Waltham, MA, USA) supplemented with 10% FBS. HL-60 cells were cultured in RPMI-1640 supplemented with 20% FBS. Cell cultures were conducted at 37°C with 5% CO_2_ in a humid incubator. Additional cell authentication has not been conducted in our laboratory. Cell passage was conducted for 26 times at most in this study. Mycoplasma test has been conducted by PCR before the use of cells. Cells used in this study were mycoplasma negative.

### Cell viability assay

Cells were plated in a 96-well plate at 10,000 cells/well and treated with or without ASP1235. Cells were incubated at 37°C for 3 days. Cell viability was measured using CellTiter-Glo2.0 Reagent (Promega, Catalog # G9243, Madison, WI, USA). Cell viability was defined as follows: cells without ADC treatment as 100% survival, medium only wells as 0% survival.

### *In vivo* anti-tumor evaluation using THP-1 xenograft mouse model

THP-1 cells were suspended in 50% PBS/50% Matrigel (BD Biosciences, Catalog # 356237, Franklin Lakes, NJ, USA) cocktail and were subcutaneously inoculated in the right frank of mice at 5 × 10^6^ cells/head. The vehicle of each drug is shown as follows: ASP1235 (20 mM Histidine/L-Histidine-HCl, 5.5% trehalose dihydrate, 0.01% Polysorbate 20, pH6.0), venetoclax (60% phosal 50PG, 30% PEG400, 10% ethanol), azacitidine (PBS). After tumors reached 100–300 mm^3^ in size, mice were randomized based on the tumor volume, and then treatment was started. ASP1235 was intravenously administrated to each mouse once per week. One day before ASP1235 treatment, each mouse received intraperitoneal injection of 20 mg/kg of nonspecific human IgG1 antibody (BIO X cell, Catalog # BE0297, Lebanon, NH, USA, RRID: AB_2687817). Venetoclax was orally administered daily at 100 mg/kg. Azacitidine was intravenously injected at 3 mg/kg for five consecutive days in the first week of treatment. Tumor volume and body weight were measured. Tumor volume was calculated by length × width^2^ × 0.5. Tumor growth inhibition rate was calculated with a formula as follows: 100 × {(tumor volume of control group) – (tumor volume of treatment group)}/{(tumor volume of control group) – (tumor volume on day 0)}. Sample size was determined based on our preliminary experiment.

### Flow cytometry

Primary peripheral blood mononuclear cells (PBMCs) isolated from chemotherapy relapsed or refractory AML patients were purchased from Cureline, Inc. (Brisbane, CA, USA) or National BioServices, Inc (Saint Petersburg, Russia). Cell lines and primary AML PBMCs were treated with human Fc block antibody (BD Biosciences, Catalog # 564220), and then subjected to cell surface and intracellular staining using stain buffer (BD Biosciences, Catalog # 554656) and Foxp3/Transcription Factor Staining Buffer Set (Thermo Fisher Scientific, Catalog # 00-5523-00), respectively. Antibodies used in this experiment are shown as follows: Alexa Fluor 488 conjugated anti-Bcl-2 antibody (BioLegend, Catalog # 658704, San Diego, CA, USA, RRID: AB_2563152), APC conjugated anti-human FLT3 antibody (Thermo Fisher Scientific, Catalog # 17-1357-42, RRID: AB_10609212), brilliant Violet 510 conjugated anti-CD45 antibody (BioLegend, Catalog # 304036, RRID: AB_2561940), Alexa Fluor 488 conjugated mouse IgG1 κ isotype control antibody (BioLegend, Catalog # 400134), APC conjugated mouse IgG1 κ isotype control antibody (Thermo Fisher Scientific, Catalog # 17-4714-82, RRID: AB_763649). Dead cells were excluded by using Zombie Violet Fixable Viability Kit (BioLegend, Catalog # 423114, RRID: not applicable). Cells were analyzed with FACS Aria III (BD Biosciences). Flow cytometry data were analyzed with Flow Jo software (BD Biosciences).

### Apoptosis assay

ASP1235 was diluted with 20 mM Histidine/L-Histidine-HCl, 5.5% trehalose dihydrate, 0.01% Polysorbate 20, pH6.0. Venetoclax and azacitidine were diluted with dimethyl sulfoxide (DMSO). THP-1 cells were seeded in a 12-well plate at 1 × 10^5^ cells/well and treated with or without ASP1235, venetoclax and azacitidine. Cells were incubated at 37°C for 2 days. Cell apoptosis was evaluated using FITC Annexin V Apoptosis Detection Kit I (BD Biosciences, Catalog # 556547). Cells were analyzed with MACS Quant analyzer (Miltenyi Biotec, North Rhine-Westphalia, Germany) and the flow cytometry data were analyzed with Flow Jo software.

### Statistical analysis

Student’s *t*-test or Dunnett’s multiple comparison test was used for the statistical analysis. The statistical analysis was conducted using GraphPad PRISM software (GraphPad Software, San Diego, CA, USA). *P*-value < 0.05 was considered significant.

## SUPPLEMENTARY MATERIALS


