# Supplementary material for: Anti-tumor effect of antibody drug conjugate ASP1235 targeting Fms-like tyrosine kinase 3 with venetoclax plus azacitidine in an acute myeloid leukemia xenograft mouse model
Source: Oncotarget. 2022 Dec 20;13:1359–68. doi: 10.18632/oncotarget.28331 (PMC9765856; doi:10.18632/oncotarget.28331)
Supplement: Supplementary file 1 [file oncotarget-13-28331-s001.pdf]

Anti-tumor effect of antibody drug conjugate ASP1235 targeting Fms-like tyrosine kinase 3 with venetoclax plus azacitidine in an acute myeloid leukemia xenograft mouse model

SUPPLEMENTARY MATERIALS

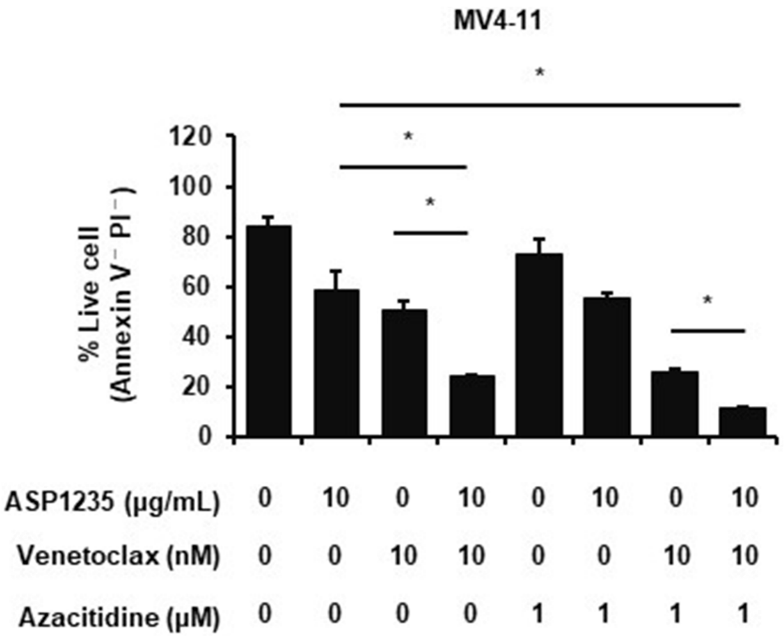

Supplementary Figure 1: ASP1235 showed enhanced cytotoxic effect with venetoclax or venetoclax plus azacitidine on MV4-11 *in vitro*. MV4-11 cells were treated with or without ASP1235, venetoclax and azacitidine at the indicated concentrations. Data are shown as mean ± standard deviation of the triplicate. * means $P < 0.05$.
